# Dynamic changes in macrophage morphology during the progression of choroidal neovascularization in a laser-induced choroidal neovascularization mouse model

**DOI:** 10.1186/s12886-023-03141-7

**Published:** 2023-10-06

**Authors:** Nana Xu, Tao Sun, Yulan Wang, Xiaowei Tong, Shiheng Lu, Fan Yang, Jing Wang, Qiyu Bo, Junran Sun, Xiaodong Sun

**Affiliations:** 1 Shanghai Eye Diseases Prevention & Treatment Center/ Shanghai Eye Hospital, Shanghai, China; 2 Department of Ophthalmology, Shanghai General Hospital (Shanghai First People’s Hospital), Shanghai Jiao Tong University School of Medicine, 100 Haining Road, Shanghai, 200080 China; 3 Shanghai Key Laboratory of Fundus Diseases, Shanghai, China; 4 Shanghai Engineering Center for Visual Science and Photomedicine, Shanghai, China

**Keywords:** Macrophage, Morphology, Choroidal neovascularization

## Abstract

**Background:**

Neovascular age-related macular degeneration (AMD) is responsible for the majority of severe vision loss cases and is mainly caused by choroidal neovascularization (CNV). This condition persists or recurs in a subset of patients and regresses after 5 or more years of anti-vascular endothelial growth factor (VEGF) treatment. The precise mechanisms of CNV continue to be elucidated. According to our previous studies, macrophages play a critical role in CNV. Herein, we aimed to determine the morphological changes in macrophages in CNV to help us understand the dynamic changes.

**Methods:**

Mice were subjected to laser injury to induce CNV, and lesion expansion and macrophage transformation were examined by immunofluorescence and confocal analysis. Several strategies were used to verify the dynamic changes in macrophages. Immunofluorescence and confocal assays were performed on choroidal flat mounts to evaluate the morphology and phenotype of macrophages in different CNV phases, and the results were further verified by western blotting and RT–PCR.

**Results:**

The location of infiltrated macrophages changed after laser injury in the CNV mouse model, and macrophage morphology also dynamically changed. Branching macrophages gradually shifted to become round with the progression of CNV, which was certified to be an M2 phenotypic shift.

**Conclusions:**

Dynamic changes in macrophage morphology were observed during CNV formation, and the round-shaped M2 phenotype could promote neovascularization. In general, the changes in morphology we observed in this study can help us to understand the critical role of macrophages in CNV progression and exploit a potential treatment option for CNV indicated by a shift in macrophage polarity.

**Supplementary Information:**

The online version contains supplementary material available at 10.1186/s12886-023-03141-7.

## Introduction

Age-related macular degeneration (AMD) is a leading cause of blindness in adults older than 55 years [1]. With the increased ageing of society, it is estimated that the number of AMD patients will increase to 288 million in 2040 [2]. There are two forms of AMD: wet or neovascular AMD and dry AMD. Although the wet form of AMD accounts for only 10 to 15% of cases, it is responsible for the majority of cases of severe vision loss [3]. Neovascular AMD is characterized by choroidal neovascularization (CNV) in older people. Although effective benefits have been achieved with anti-VEGF treatment, choroidal neovascularization still progresses or regresses in some cases after 5 or more years of treatment [1, 2, 4, 5]. Consequently, studies have show that blood inflammatory indices, angiogenesis-associated chemokine receptors such as IL-10, and CCR2^+^ and CX3CR1^+^ nonclassical monocytes were increased in refractory nAMD patients, suggesting that the poor therapeutic outcomes in the late stage may be related to macrophage overactivation and abnormal differentiation, but the specific mechanism is still unknown [6, 7]. The precise mechanisms contributing to CNV continue to be elucidated.

In physiological and pathological conditions, the innate immune system plays a critical role in pathological angiogenesis, including endothelial cell proliferation, migration and vessel anastomosis [8]. The links between macrophages and angiogenesis have been defined in several pathophysiological diseases, such as eye disorders, cancers with vigorous vessels and atherosclerosis [9–11]. Macrophages have garnered increasing interest in the pathogenesis of AMD. Macrophages are a diverse group of cells originating from the mononuclear phagocytic lineage. They are highly plastic, exhibit dramatic phenotypic changes in response to various stimuli and exist in a multitude of subpopulations [12]. Depending on the tissue context, macrophages can be classically (M1) or alternatively (M2) activated. Numerous studies have shown that macrophages that respond to injury in the early stage are an inflammatory phenotype (M1), and later, M1 cells shift towards an anti-inflammatory, proangiogenic, profibrotic, and wound healing phenotype (M2) [13]. However, macrophages are highly plastic, and M1 macrophages can differentiate into the M2 phenotype in numerous diseases, such as chronic inflammatory diseases and cancers [14]. Likewise, M2 macrophages can transition to M1 cells when treated with IFN-ɑ or antibodies against CD40 [15], suggesting that the M1 and M2 macrophage phenotypes are reversible according to distinct immune environments. Studies on peripheral blood mononuclear cells from AMD patients showed that macrophage-derived miR-150 was significantly elevated, which was associated with pathologic angiogenesis in a VEGF-independent manner [6]. Consistently, it was also demonstrated that the switch in macrophage phenotype from M1 to M2 was responsible for the increase in CNV in an old mouse model [16]. A similar situation [17] was observed in nAMD patients, in which alternatively activated ocular macrophages (M2) were the majority in the advanced stage of CNV [18, 19]. Thus, examining dynamic changes in macrophage polarity could yield therapeutic outcomes in the treatment of neovascular diseases.

In our previous studies, the depletion of macrophages alleviated CNV progression in a mouse model, but the role of M1 and M2 macrophages was unclear. Furthermore, we inhibited M2 macrophages with a specific antibody, which alleviated CNV [20]; however, the dynamic changes in macrophage differentiation are still unclear, which indicates a new basis for CNV treatment. In the present study, we used confocal immunofluorescence analysis to examine macrophage morphology in different CNV phases. Interestingly, we found that macrophages appeared immediately in response to laser injury in a mouse model and gradually infiltrated the CNV lesions. Then, the morphology of macrophages that initially appeared around the laser spot changed, and the morphology was distinct within or away from the vigorous neovascular area. With the growth of CNV, we found that macrophages gradually infiltrated the lesion and underwent morphological changes from branching to round-shaped. Furthermore, we identified these round-shaped macrophages as M2 macrophages that were restricted within or around the neovascular areas. Collectively, our data showed the dynamic characteristics of macrophage morphology in choroidal neovascularization, which may point to M2 polarity drift as an intervention target for the treatment of CNV. These data may also determine distinct immune environments around CNV lesions, which provides novel knowledge of macrophage polarization and critical treatment targets for CNV.

## Methods

All methods are reported in accordance with ARRIVE guidelines.

### Laser-induced mouse CNV model

The animals were treated according to the guidelines of the ARVO Statement for the Use of Animals in Ophthalmic and Vision Research. The experimental procedures were approved by the Institutional Animal Care and Use Ethics Committee of Shanghai Jiao Tong University (Shanghai, China). C57BL/6 J male mice (aged between 6 and 8 weeks and approximately 20 g, supplied by the Laboratory Animal Center at the Shanghai First People’s Hospital) were included. After the application of tropicamide (Santen, Osaka, Japan) for pupil dilatation, the animals were anaesthetized with an intraperitoneal injection of 1% pentobarbital sodium (0.1 mL/10 g body weight) (Guge Biotech, Wuhan, China). A glass coverslip was lubricated with loxacin eye ointment (Xing Qi Pharmaceutical Companies, Shenyang, China), and four laser spots were distributed around the optic nerve head with an argon laser (110 mW, 100 ms, 50 μm, OcuLight Infrared Laser System 810 nm, Iridex Corp., Mountain View, CA, USA). The appearance of a grey bubble indicative of the rupture of Bruch’s membrane was observed. If retinal bleeding occurred, the animal was eliminated. The eyes were enucleated at different time points [20, 21].

### Immunofluorescence analysis

Immunofluorescence assays were performed on choroidal flat mounts. Briefly, after being fixed, the samples were blocked with 0.3% Triton X-100 and 5% goat serum albumin (Beyotime, Shanghai, China) in PBS for 1 h at room temperature. The tissues were immunostained with primary antibodies against F4/80 (1:500; CST, Beverly, MA, USA), Ym-1 (1:500; Stem Cell Technology, Vancouver, Canada) and isolectin (1:1000; Santa Cruz Biotechnology, Santa Cruz, CA, USA) overnight at 4 °C, washed three times with PBS, and then stained for 45 min at 37 °C with Alexa Fluor 594- and 488-conjugated secondary antibodies (1:1000; Proteintech, Chicago, IL, USA). The nuclei were stained with 4,6-diamidino-2-phenylindole. Images were visualized using a fluorescence microscope (Olympus, Center Valley, PA, USA) and a Leica TCS SP8 confocal laser scanning microscope (Leica TCS NT, Wetzlar, Germany) [20].

### Reverse transcription–polymerase chain reaction

RNA extraction was performed according to the RNA Simple Total Kit protocol (Tiangen Biotech, Beijing, China). A NanoDrop 2000c spectrophotometer (Thermo Fisher Scientific, Wilmington, DE, USA) was used to quantify the RNA samples. RT Master Mix (Takara Bio, Inc., Dalian, China) was used for cDNA synthesis. The samples were run on a real-time PCR detection system (Eppendorf, Hamburg, Germany) with a SYBR green-based PCR method for mRNA expression analysis. All reactions were prepared in triplicate. Target gene expression was analysed using the comparative cycle threshold (CT) method [22].

### Western blot analysis

Samples were subjected to 10% SDS–PAGE, transferred to a polyvinylidene fluoride membrane, blocked with blocking buffer (Tris-buffered saline Tween-20 [TBST], containing 5% nonfat dry milk) for one hour at room temperature and incubated with primary antibodies against Arg-1 (1:1000; Abcam, Cambridge, MA, USA), iNOS-1 (1:1000; Stem Cell Technology), and GAPDH (1:1000, Cell Signaling Technology, Beverly, MA, USA) overnight. The membranes were then washed three times with TBST and probed with horseradish peroxidase-conjugated secondary antibodies (1:2000, Proteintech) for one hour at room temperature. The membranes were then washed with TBST and exposed to a molecular imaging system (Amersham Imager 600, GE Healthcare, Buckinghamshire, UK) [20].

### Statistical analysis

The data are expressed as the mean ± SE. Each experiment was performed in triplicate. Differences among groups were determined using one-way ANOVA. For two-group analysis, Student’s t test (GraphPad Prism, San Diego, CA, USA) was used. The 0.05 level of probability was used as the criterion of significance.

## Results

### The position of infiltrated macrophages changed at distinct time points after laser injury in the CNV mouse model

**Fig. 1:**
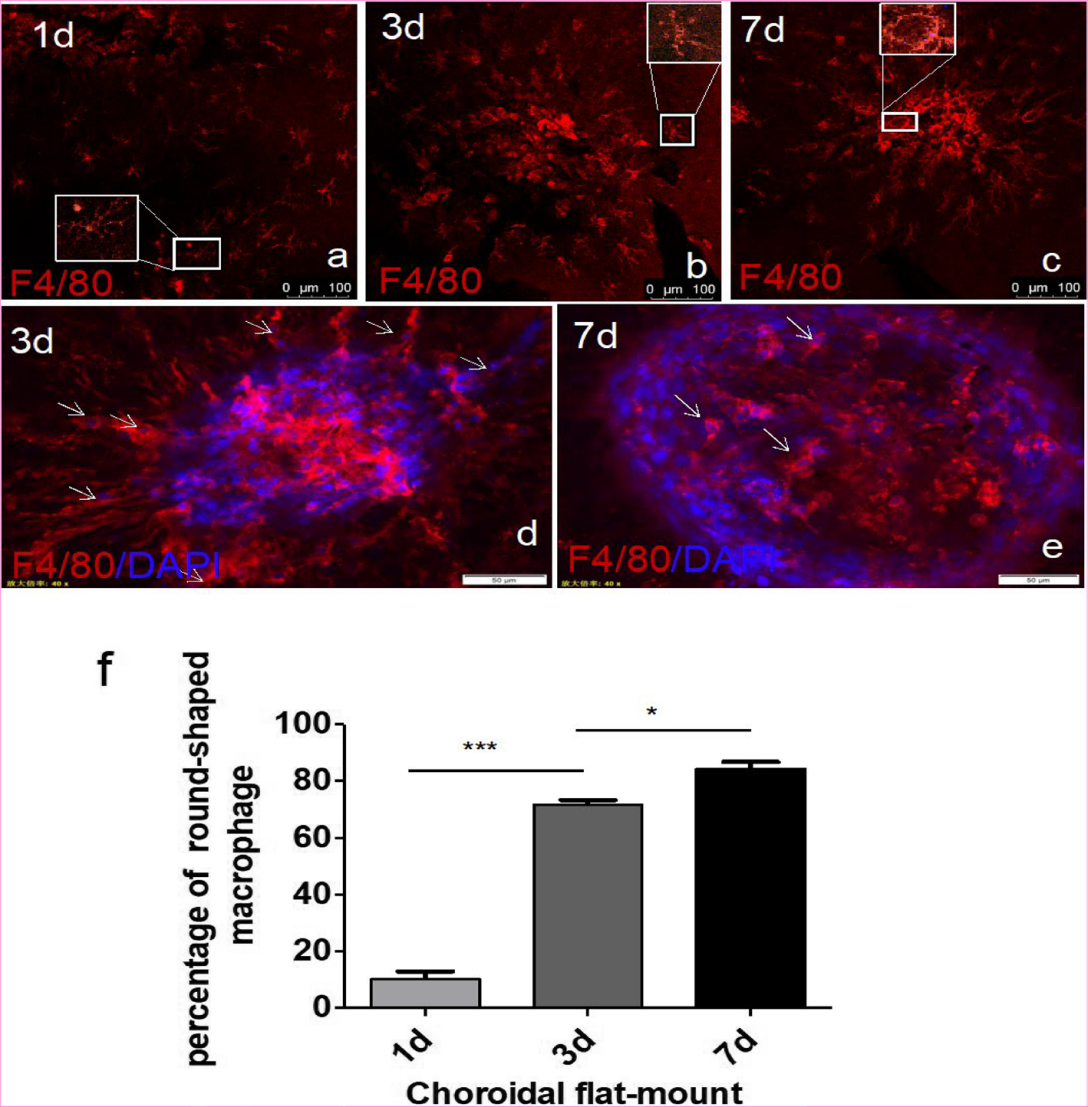
**a-e** The location and morphology of infiltrated macrophages were different at distinct time points (1, 3, and 7 days) after laser injury in the CNV mouse model. **d-f** Macrophages gradually became round as neovascularization progressed. *P < 0.05; **P < 0.01; ***P < 0.001 via Student’s t tests. Scale bar: **a**-**c** 100 μm, **d**, **e** 50 μm. (*n* = 3 to 5 per group)

**Fig. 2:**
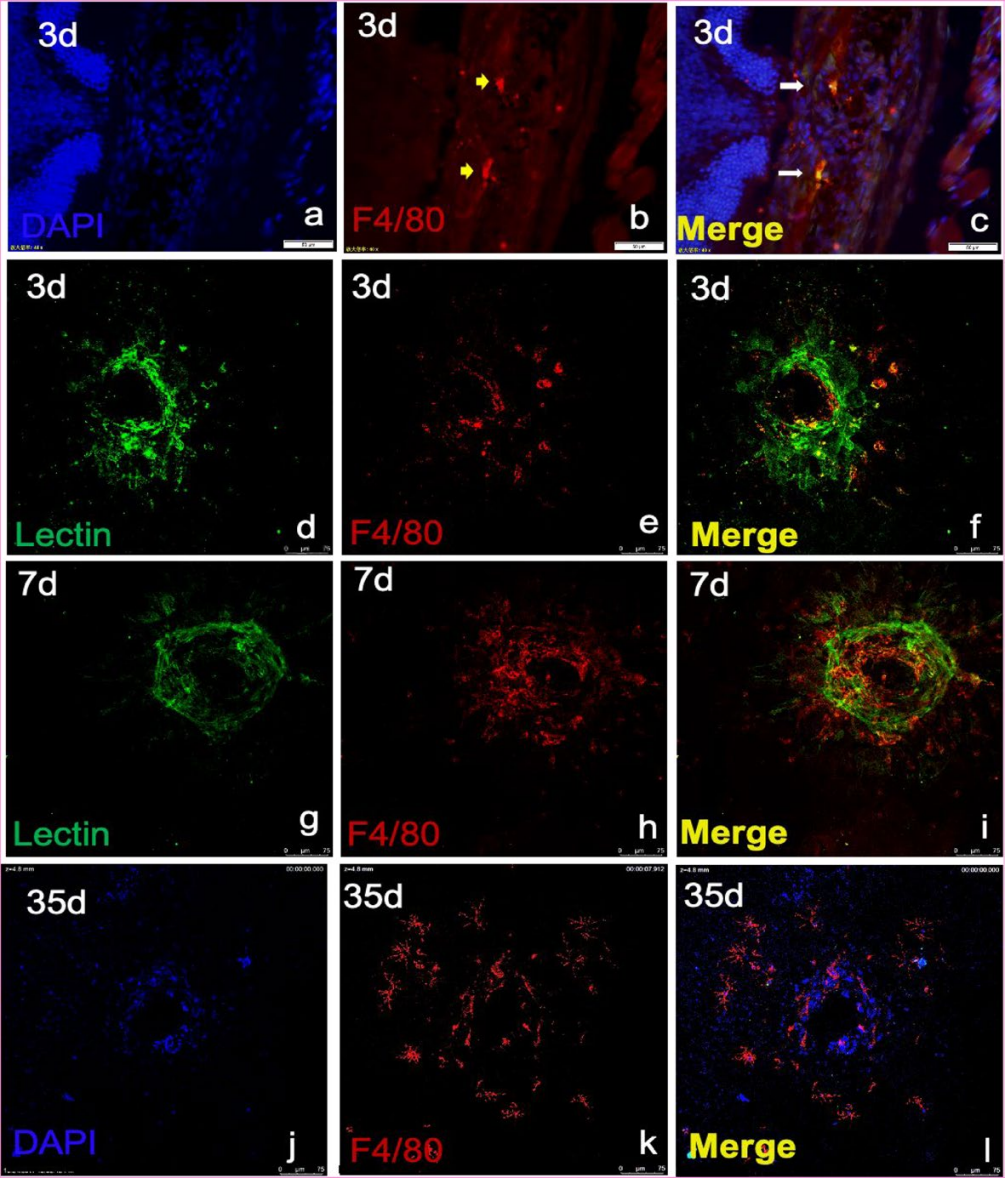
**a-i** Macrophages infiltrated the core area of the neovascularization lesion during the progression of CNV (Days 3, 7, and 35). Scale bars: **a**-**c**: 50 μm and **d**-**l**: 75 μm. (*n* = 3 to 5 per group)

To examine the role of macrophages in the progression of neovascularization, we performed confocal immunofluorescence localization analysis of choroidal flat mounts to identify the location of macrophages at different time points after laser injury. On the first day after laser injury, many macrophages emerged around the injury sites; however, no macrophages were present inside the injury sites (Fig. 1a). On the third day, increased macrophages appeared around the injury sites where neovasculature began (Figs. 1b, d and 2d-f). Gradually, macrophages significantly infiltrated the core of the CNV lesion, while other macrophages migrated from a distance (Figs. 1b, d and 2a-c). In conclusion, we examined different stages of CNV formation and identified changes in macrophage position during the progression of CNV, which suggested the critical role of macrophages in CNV formation.

### Dynamic changes in macrophage morphology during the formation of CNV

To further examine the transformation of macrophages during the progression of CNV, we conducted confocal immunofluorescence analysis of choroidal flat mounts to further identify the morphological changes in macrophages in different phases after CNV induction. On the first day after laser injury, many macrophages emerged around the injury sites, most of which were branching, and only 10% were round (Fig. 1 F). On the third day, additional macrophages infiltrated the injury sites where neovasculature began to develop. Interestingly, up to 70% of macrophages in the neovascular region were round, while the distant macrophages were branching (Fig. 1b, d, f). On the seventh day, there were up to 85% round macrophages in the CNV area (Fig. 1c, e, f). These results showed the marked deformation of infiltrated macrophages from branching to round, which was consistent with the growth of CNV. These findings suggested that macrophages participate in neovascularization with distinct states and that the ratio of round macrophages increased with time.

### Round-shaped macrophages were alternatively activated according to the dynamic microenvironment and were conducive to the formation of CNV

**Fig. 3:**
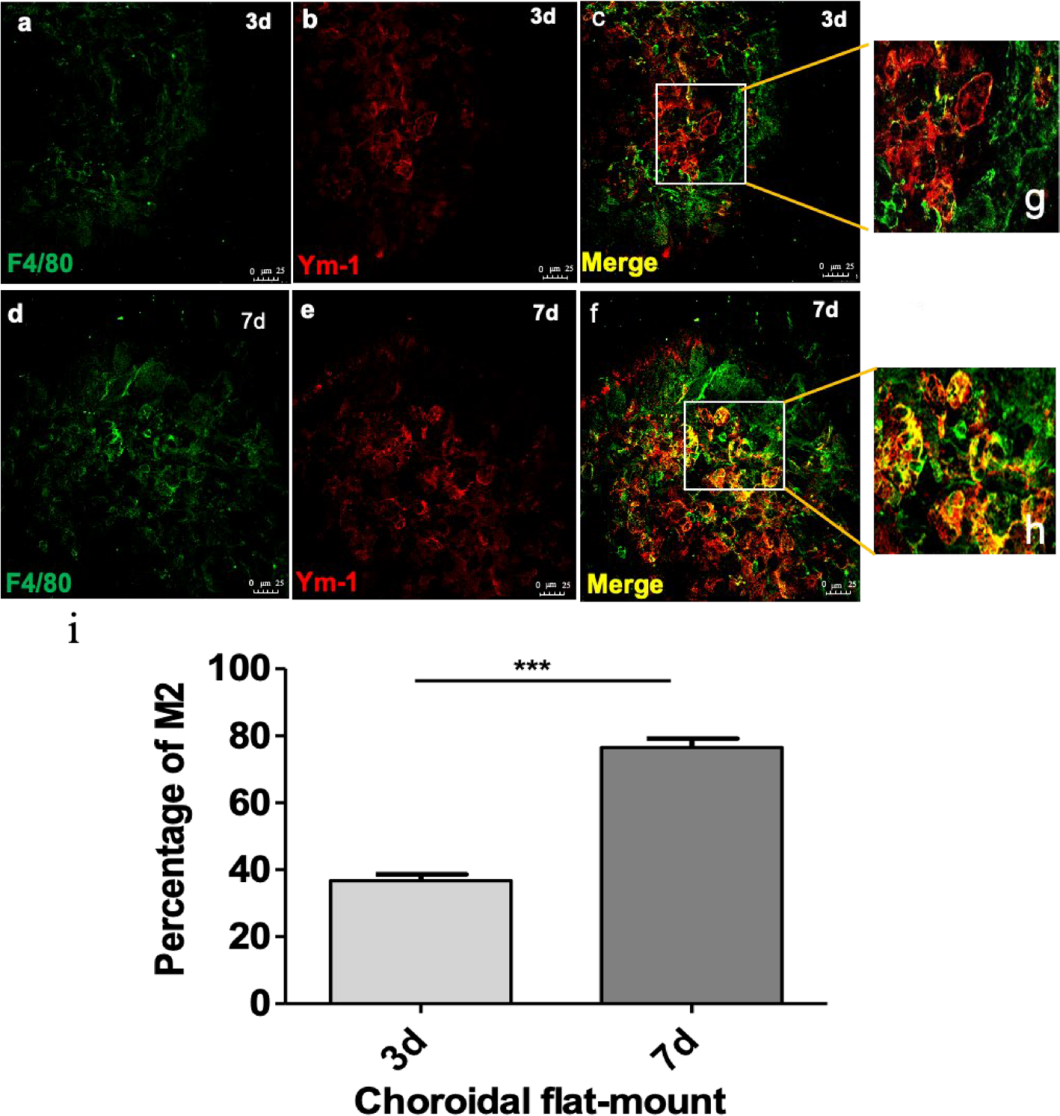
**a-h** Macrophages infiltrated the core area of the neovascularization lesion. **i** Macrophages gradually became round (M2) from the 3rd to the 7th day. *P < 0.05; **P < 0.01; ***P < 0.001 via Student’s t tests. Scale bar: **a**-**f** 25 μm. (*n* = 3 to 5 per group)

To determine the type of macrophages present in different regions and phases of CNV, we performed immunofluorescence analysis to evaluate the phenotypes of dynamically changing macrophages. According to our previous study [20, 23], M2 macrophages play a critical role in CNV progression, and so we first identified the presence of M2 macrophages (Ym-1^+^). In contrast with the first day, on the third day after CNV induction, when neovascularization began to be vigorous (Fig. 2d-f), the number of macrophages increased, and most macrophages infiltrated into the core of the CNV area and become round-shaped, 38% of which were identified as M2 cells (Ym-1^+^) (Fig. 3a-c, g, i). On the seventh day, the formation of the neovascularization was robust, and the percentage of round-shaped macrophages increased to almost 80%, which suggested a critical role of M2 macrophages in CNV (Figs. 2g-i and 3d-f, h, i). On the thirty-fifth day of the late stage, when CNV progressed to fibrosis, we also observed branching macrophages around the lesion and round-shaped macrophages in the core of the lesion (Fig. 2j-l).

### The M1/M2 shift can be further identified by expression profiling during neovascularization

**Fig. 4:**
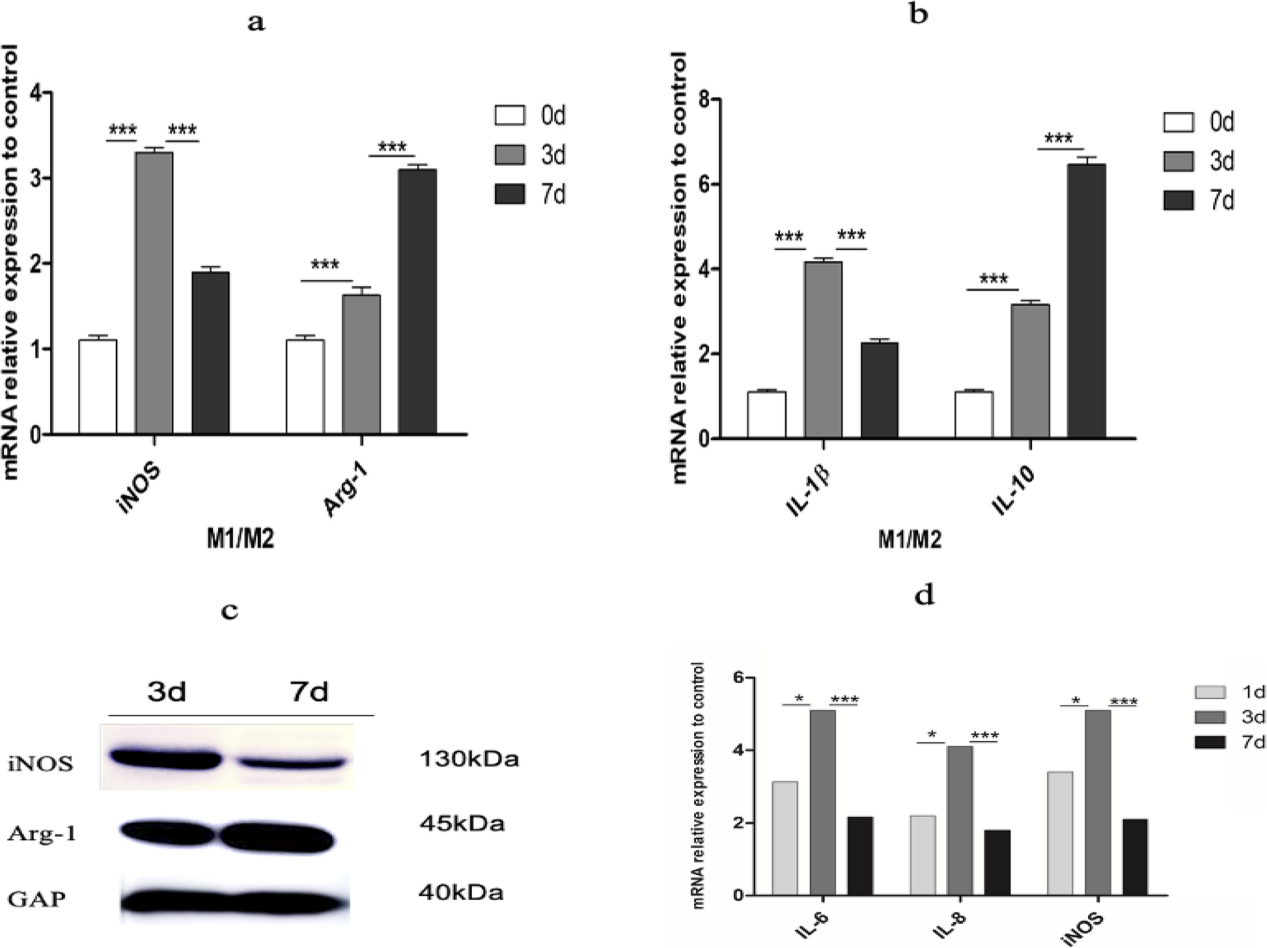
**a**,** b** iNOS and IL-1β (M1 profile) expression increased after laser injury until the 3rd day and began to decline on the 7th day. Arg-1 and IL-10 (M2 profile) expression were gradually increased until the 7th day. **c** iNOS protein expression decreased from the 3rd day to the 7th day, while Arg-1 expression was upregulated. **d** Increased choroidal and retinal inflammatory gene expression in the CNV mouse model: RNA was isolated from RPE/choroids from the control and CNV groups 1 day, 3 days and 7 days after CNV induction to measure the mRNA expression of proinflammatory cytokines by RT–PCR. *P < 0.05; **P < 0.01; ***P < 0.001 versus control (*n* = 3 to 5 per group)

To further determine the role of macrophages in the formation of CNV, we performed PCR and western blotting to evaluate the expression profiles of M1 and M2 macrophages. M1 macrophages are a classical inflammatory population that expression interleukin (IL)-1β and iNOS, while M2 macrophages are associated with neovascularization stimulated by specific cytokines, including interleukin 10 (IL-10) and Arg-1 [24]. The production of M1 markers, such as IL-1β and iNOS, increased robustly after laser injury, continued to increase until the 3rd day and began to decline on the 7th day (Fig. 4a-c). These M1 macrophages had strong proinflammatory potential to recruit more macrophages and neutrophils after tissue injury by upregulating the expression of IL-6, IL-8 and iNOS on Days 1 and 3 after CNV induction (Fig. 4d). In contrast, the M2 profile expression was gradually elevated until the 7th day, which was to the opposite of the M1 profile (Fig. 4a-c). These M2 macrophages could rescue acute inflammation and contribute to tissue repair and neovascularization, which was reflected by the decreased expression of IL-6, IL-8 and iNOS on the 7^th^ day (Fig. 4d). Interestingly, these reverse expression curves were consistent with the morphological changes in macrophages. Based on these findings, we concluded that M1 macrophages emerged as branching cells during the inflammation stage, which was conducive to movement and adhesion to injured tissues, and secreted proinflammatory factors such as IL-1β, IL-8, IL-6 and iNOS to recruit more macrophages. In the late stage of tissue injury, macrophages became round and secreted cytokines such as IL-4, IL-10, and VEGF-A to assist in tissue repair, such as neovascularization and fibrosis, in nAMD. These findings demonstrated the plasticity of retinal macrophages, which underwent programmatic changes from a homeostatic state to disease response during the course of CNV.

## Discussion

Macrophages are important regulators of age-related degenerative diseases and inflammatory-related diseases [25]. We have provided evidence indicating that macrophages participate in angiogenesis with distinct morphologies in the early and late stages of CNV. Substantial evidence has shown that RPE-derived VEGF is the main cause [26] of CNV, but VEGF can also be induced by other mechanisms; for example, macrophages are an important source, as indicated in our previous study on CNV [20].

Macrophages play critical roles in the innate immune system in both physiological and pathological conditions and have been shown to play a determinant role in vascular remodelling [27]. Depending on the tissue microenvironment, macrophages can be classically (M1) or alternatively (M2) activated [19]. Macrophage subpopulations are characterized by specific markers and produce different cytokines and receptors [28]. M1 macrophages are proinflammatory and antiangiogenic and have elevated expression of inducible nitric oxide synthase (iNOS), TNF-α, IL-12, IL-6, IL- 1β and matrix metalloproteinase-9 (MMP-9). In contrast, M2 macrophages are proangiogenic, promote wound healing and are characterized by decreased M1 marker expression and increased levels of IL-10, CD163, and TGF-β [29].

In our study, the coexistence of macrophages with different morphologies was observed, which reflected the complex microenvironment during CNV progression. Macrophage morphology changed from branching to round, and the phenotype shifted to the alternatively activated (M2) state, which was consistent with a study showing that the dysregulation of cholesterol metabolism in nAMD could polarize macrophages to the proangiogenic M2 phenotype [9]. In our previous study, M2 macrophages were shown to promote neovascularization [20], and the changes in morphology we observed in this study can further help us to understand the crucial role macrophages play in CNV.

In early studies, it was suggested that macrophages were plastic according to dynamic pathological conditions [17]. However, these studies did not examine the morphology of macrophages in distinct stages. In this study, we observed that branching macrophages emerged in the early stage of CNV, which was consistent with previous studies showing that the activation of innate immunity was often an early event in the disease process [14]. Subsequently, branching macrophages became round, which was shown to be the M2 phenotype to some extent. We hypothesized that injury and pathological stimulation immediately activated macrophages originating from the tissue and circulatory system to be branching, and this morphology is conducive to moving to the focal lesion. When the cells arrive, the branching macrophages become round and are a major source of cytokines, such as VEGF, IL-10, IL-1, IL-6, IL-8, and IL-12, to mediate inflammation and neovascularization [23]. Consequently, we can estimate the functions of macrophages by examining the morphological transformation during pathological CNV, which can provide a basis for macrophage-focused diagnostic and therapeutic strategies. However, the exact mechanism and molecules associated with macrophage morphological plasticity still need to be examined. Collectively, our data showed dynamic morphologic characteristics of macrophages during the choroidal neovascularization process, which may point to M2 polarity drift as an intervention target for the treatment of CNV.

### Supplementary Information

**Additional file 1.**

## Data Availability

The datasets generated and/or analysed during the current study are available from the corresponding author upon reasonable request.
