# Supplementary figures and images for: Dynamic changes in macrophage morphology during the progression of choroidal neovascularization in a laser-induced choroidal neovascularization mouse model
Source: BMC Ophthalmol. 2023 Oct 6;23:401. doi: 10.1186/s12886-023-03141-7 (PMC10559478; doi:10.1186/s12886-023-03141-7)

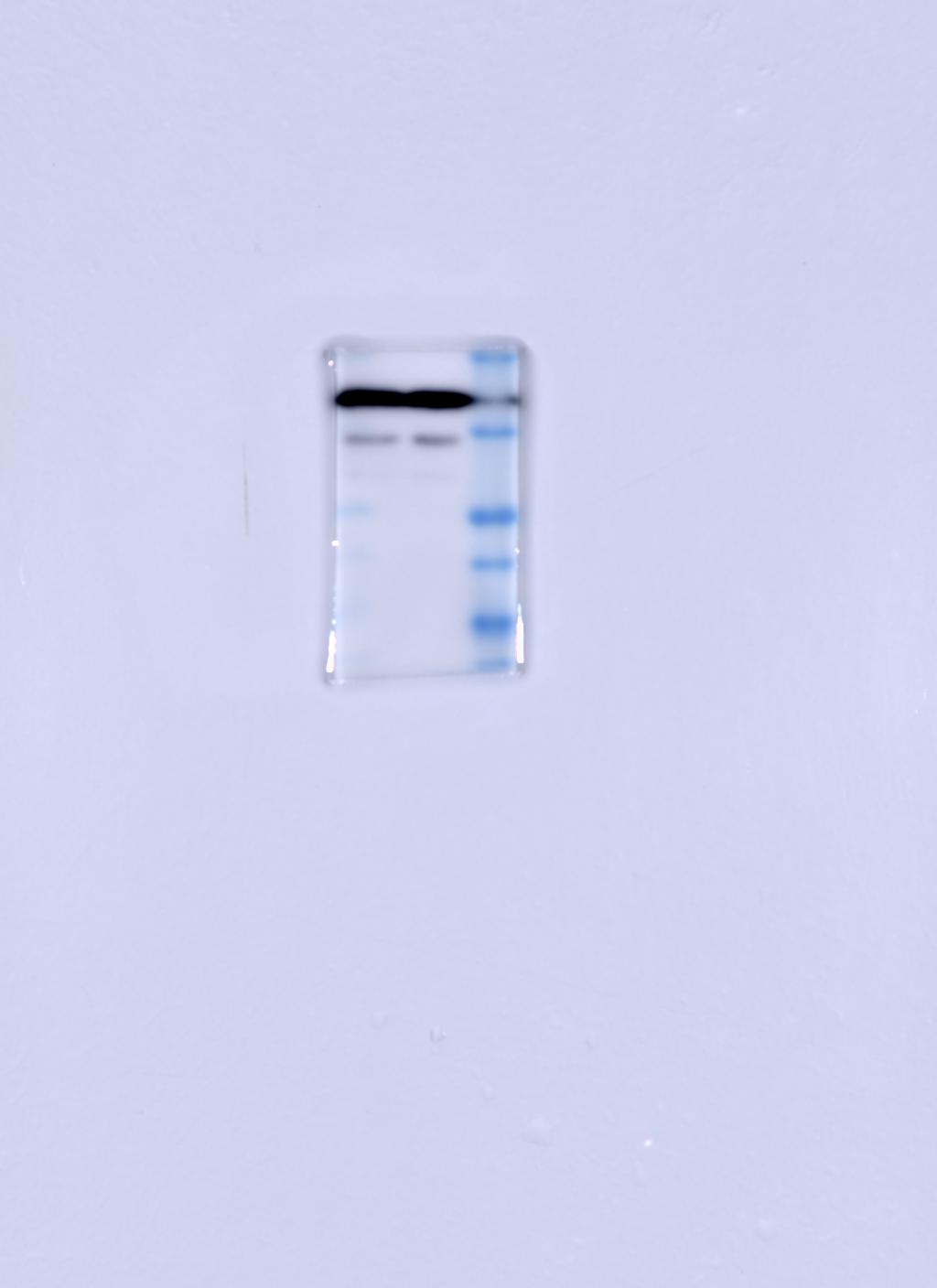

Supplement: Supplementary file 1 — Additional file 1. [file 12886_2023_3141_MOESM1_ESM.zip › supplmentary material for fig4/Fig 4c gap.jpg]

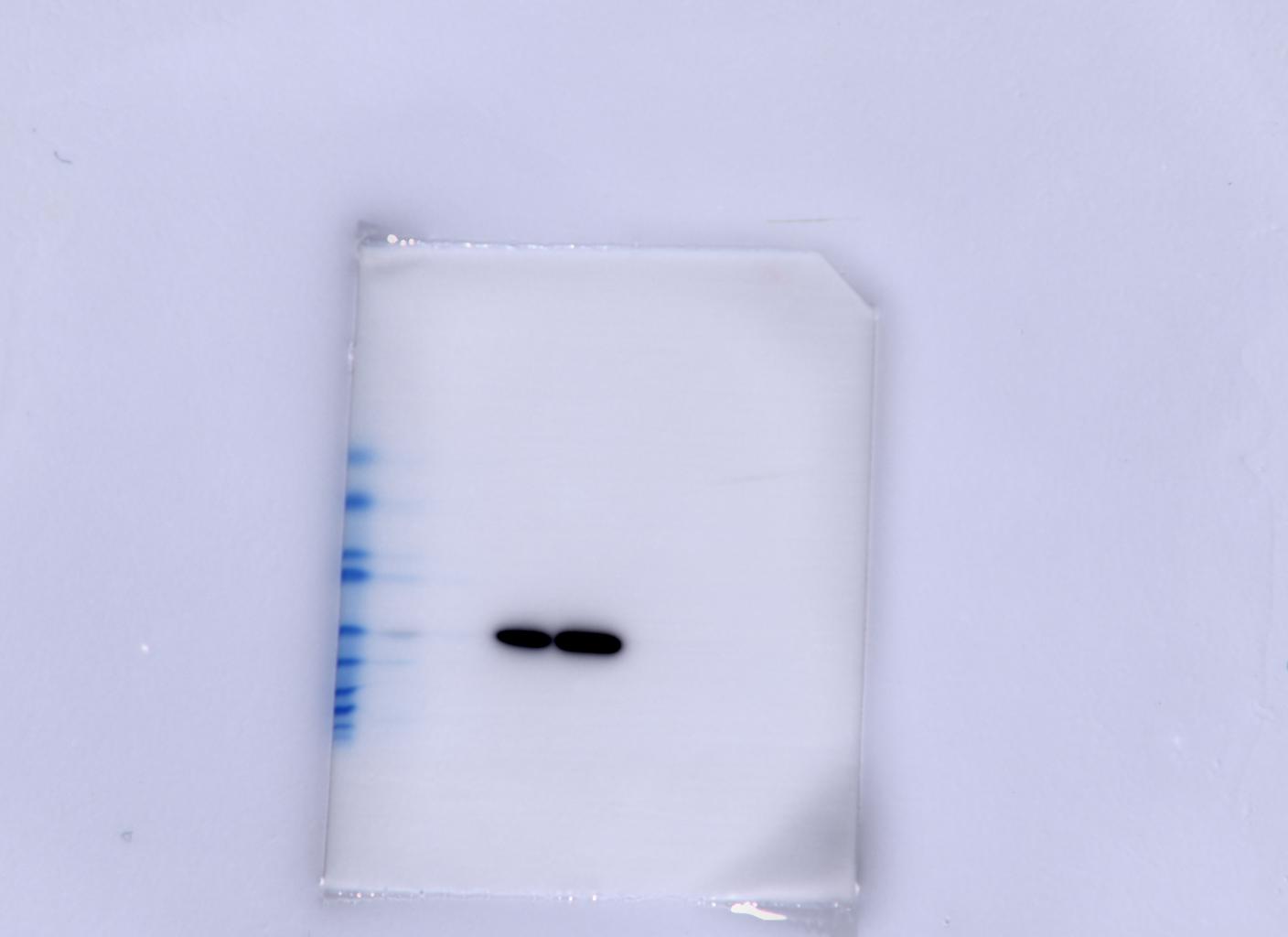

Supplement: Supplementary file 1 — Additional file 1. [file 12886_2023_3141_MOESM1_ESM.zip › supplmentary material for fig4/Fig 4c arg1.jpg]

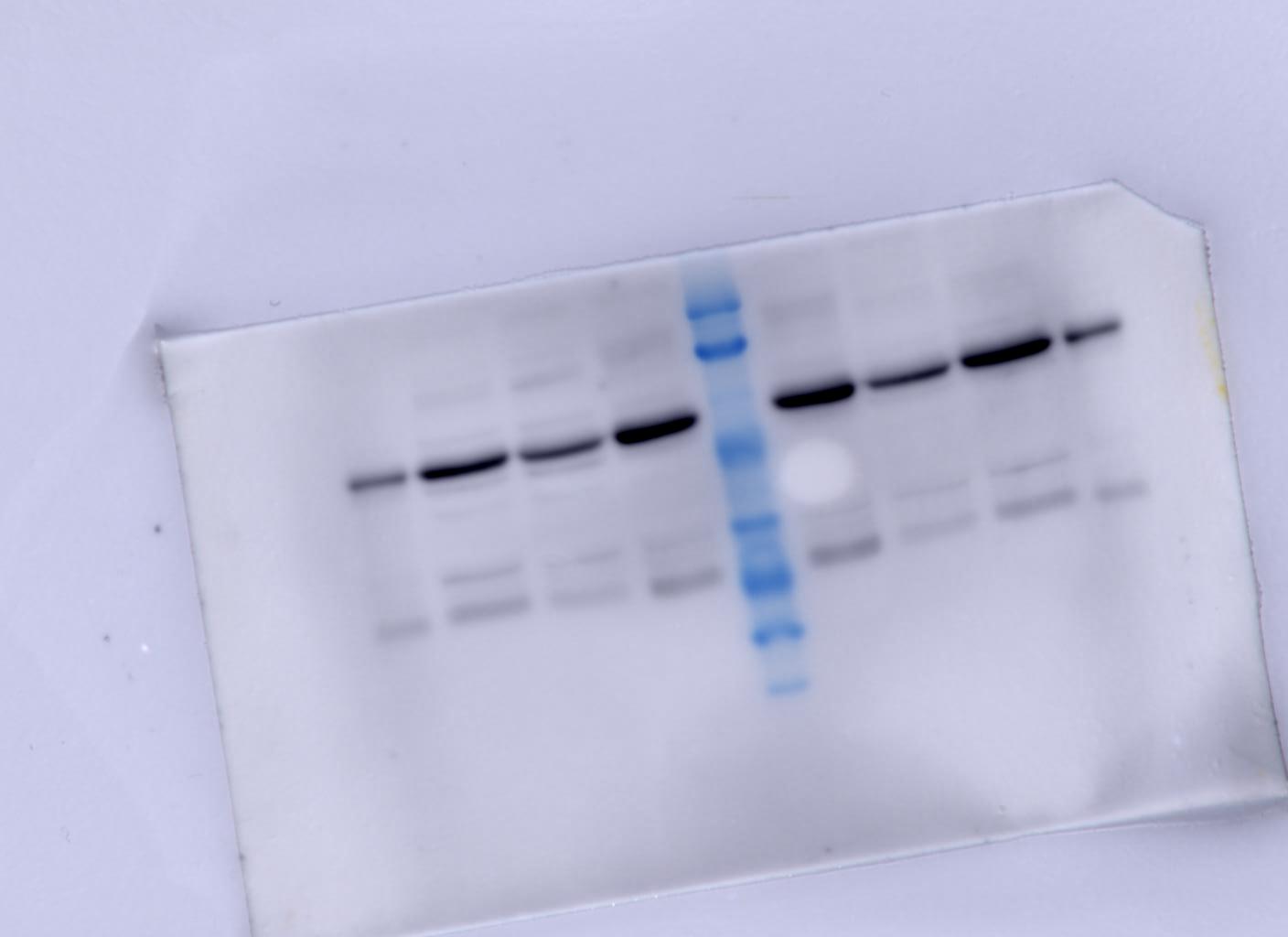

Supplement: Supplementary file 1 — Additional file 1. [file 12886_2023_3141_MOESM1_ESM.zip › supplmentary material for fig4/Fig 4c iNOS.jpg]
